# Sand and grass surfaces are equally effective in promoting positive adaptations in the sprint performance of elite young soccer players

**DOI:** 10.5114/biolsport.2023.123324

**Published:** 2023-02-03

**Authors:** Lucas A. Pereira, Renan F. H. Nunes, Tomás. T. Freitas, Carlos A. Paes, Juan H.S. Conde, Luiz F. Novack, Thiago Kosloski, Rodrigo L.P. Silva, Paulo H.S.M. Azevedo, Irineu Loturco

**Affiliations:** 1 NAR – Nucleus of High Performance in Sport, São Paulo, Brazil; 2 Department of Human Movement Sciences, Federal University of São Paulo, São Paulo, Brazil; 3 Coritiba Football Club, Curitiba, Brazil; 4 UCAM Research Center for High Performance Sport, UCAM Universidad Católica de Murcia, Murcia, Spain; 5 Facultad de Deporte, UCAM Universidad Católica de Murcia, Murcia, Spain; 6 University of South Wales, Pontypridd, Wales, United Kingdom

**Keywords:** Athletic performance, Athletes, Muscle power, Plyometrics, Football, Team sports

## Abstract

This study compared the effects of two sprint-jump training programmes, performed on either sand or grass surfaces, on the sprint and jump performance of elite young soccer players over an 8-week training period. Fifteen under-20 soccer players were randomly allocated to the sand (n = 7) or grass (n = 8) group. Athletes performed 12 training sessions, comprising vertical and horizontal jump exercises, and linear and change-of-direction (COD) sprint drills. Pre- and post-measurements were completed in the following order: vertical jump, sprint speed at 10 m and 17 m, curve sprint (CS), and modified Zigzag COD tests. Between-group differences were determined using a two-way ANOVA with repeated measures and effect sizes (ES). No improvements in jump performance were found in either group. Significant increases were observed in the sand group for acceleration in 0–10 m and for 10- and 17-m linear sprint velocity (ES = 1.15, 1.16, and 1.81, respectively; P < 0.05). In contrast, no significant differences were detected for acceleration and linear sprint velocity in the grass group, comparing pre- and post-tests (ES ranging from 0.01 to 0.47; P > 0.05). Both sand and grass groups revealed similar increases in the CS and COD velocities after the training period (ES ranging from 0.98 to 1.93; P < 0.05). In conclusion, sprint-jump training programmes performed on both grass and sand surfaces elicited significant improvements in CS and COD performances, whereas acceleration and linear sprint velocity increased only in the sand group, after a short-term training period. The sand training surface was proven to be a practical strategy to improve sprint performance in all its forms in soccer players, which is of great interest and importance for coaches and sport scientists working in elite soccer.

## INTRODUCTION

Soccer is an intermittent team sport characterized by short-duration, highly demanding activities interspaced with low- and moderate-intensity periods [1–3]. From a general perspective, the multiple physical tasks performed by soccer players during matches rely on aerobic metabolism and on high levels of speed and power [3]. Indeed, previous studies have already shown that sprinting and jumping are the most frequent actions preceding goal scoring situations and that, importantly, sprint distance and number of maximal sprints have increased substantially in professional soccer over the last few years [1, 2]. As a consequence, special attention has been paid to the proper and efficient development of neuromuscular qualities in elite soccer players [3–5].

Several training strategies have been proposed and used to improve speed and power capacities in team-sport athletes from different performance levels and age categories [3–5]. Recent surveys conducted with soccer practitioners working in national leagues from different countries revealed that “plyometrics” and “maximum speed sprinting” were the most frequently used training methods for increasing sprinting speed and power output in elite soccer players [4, 5]. In general, these training strategies are executed on “harder surfaces” (e.g., gym floor or grass) [6] based on the assumption that “softer surfaces” (e.g., sand) could dissipate a substantial amount of ground reaction forces (and elastic energy) during plyometric activities [7–10], thus affecting movement velocity, force, and power production [6]. Therefore, soft surfaces might not be “optimal” to activate and potentiate the stretch-shortening cycle [7–10] and may contribute to higher energy expenditure when compared to sprint or jump tasks performed on harder surfaces at similar speeds or heights, which in turn may potentially impact the subjective perception of effort [6, 11, 12]. However, somewhat surprisingly, training on a sand surface has been proven to be as efficient as training on harder surfaces in terms of speed-power development (e.g., large effect sizes [> 1.0] and significant changes [*P* < 0.05] in jump and sprint performance) [6]. In fact, it has been observed that sand training may lead to higher motor unit recruitment in the muscle groups involved in some specific motor tasks (when compared to harder surfaces), which indicates that this training strategy might act as an “alternative way” to increase training load and thus promote positive changes in physical performance [6]. Despite these preliminary indications, the limited level of evidence regarding the effectiveness of this training method, specifically in highly trained athletes, precludes more robust conclusions on this issue.

Another crucial point to consider when implementing speed training strategies for soccer players is the importance of providing stimuli involving not only linear, but also other forms of sprinting (i.e., change-of-direction [COD], and curved sprints). A systematic review examining the number and nature of high-intensity activities executed during soccer matches revealed that each player performs between 7 and 61 linear sprints and > 300 directional changes with different cut angles, turns, and “swerves” (i.e., short and fast multi-directional movements) per match [13]. Moreover, a recent study reported that the vast majority of sprints (i.e., 80%) performed during soccer matches are curvilinear [14]. In this regard, it has been suggested that, to appropriately improve multidirectional speed qualities, mixed training approaches involving decelerations, accelerations and COD tasks should be implemented [4, 15, 16]. Nevertheless, the impact of implementing these mixed training strategies on the speed-related performance of team-sport athletes is still unknown, especially when considering the potential adaptations associated with different surface types (e.g., sand, or harder surfaces).

Developing practical training strategies able to maximize speed and power output in elite soccer players is unquestionably of great interest for practitioners [4, 5]. The use of plyometric drills combined with maximal sprint bouts, executed on either soft or hard surfaces, seems to be a very viable alternative. However, the current evidence [6] does not allow us to draw any firm conclusion regarding the superiority of one surface over the other. Therefore, the purpose of this study was to investigate and compare the effects of two different training programmes (i.e., mixed plyometric and sprint training strategies performed on sand or grass surfaces) on the jump and speed-related performance (assessed by linear, curvilinear, and COD speed tests) of elite young soccer players. Considering previous investigations [6, 8] on this topic, we hypothesized that both training strategies would be equally effective to elicit positive changes in the physical performance of the athletes.

## MATERIALS AND METHODS

### Participants

Eighteen elite young soccer players (18.5 ± 0.6 years; 71.7 ± 5.0 kg; 178.6 ± 7.5 cm) from the same club, playing in the 1^st^ division of the under-20 Brazilian National Championship, participated in this study. Athletes were randomly allocated to two training groups, as follows: the “sand training group” (n = 9); and the “grass training group” (n = 9). Player names were entered in order of lowest to highest 17-m sprint times, by an independent researcher, in a customized spreadsheet, and grouped in pairs according to their baseline results. Subsequently, the group allocation of each pair was determined by tossing a coin. Three athletes did not complete all training sessions and were excluded from the analysis. Thus, the final dataset included 15 athletes (7 athletes in the sand group and 8 athletes in the grass group). The research was approved by the local Ethics Committee (registration number 5.200.656) and all participants and their legal guardians signed an informed consent form prior to participation in the study.

### Study Design

This parallel, two-group, quasi-randomized study was designed to test the effects of two mixed-training programmes, performed on a sand or grass surface, on jump and sprint performances of elite young soccer players during an 8-week inter-season training period. Athletes were tested after the São Paulo Cup of Junior Soccer Players and before the Parana State Championship (under-20 category). The training content followed by the athletes during the intervention period is presented in Table 1. Except for the distinct training surfaces, players from both groups followed the same training routines throughout the study, which consisted of 12 sand or grass training workouts, 8 resistance training sessions, 28 technical and tactical training sessions, and 5 friendly matches. The training sessions were performed simultaneously by both groups at the soccer club’s training facilities, with the sand and grass fields located next to each other. Table 2 shows the typical weekly training schedule of the players across the 8 weeks. All athletes were previously familiarized with the training and testing routines. The physical tests were conducted in the following order: squat and countermovement jump (SJ and CMJ), sprinting speed at 10 m and 17 m, curve sprint test (CS), and a modified Zigzag COD test. For the sake of consistency and to facilitate data interpretation, the distance of the linear and multidirectional sprint tests was standardized according to the previously validated CS test [17], which is performed on the “official arc of the soccer area” (i.e., 17 m). All tests were conducted at the same time of day, and the temperature (~20°C vs. ~18°C) and wind velocity (~3 km · h^−1^) were similar between pre- and post-assessments. Prior to measurements, a general and specific warm-up was performed involving light running (rating of perceived exertion [RPE] of 3–4 on a 1–10 scale [18]) for 10 min followed by 3 submaximal jumps, and 2 submaximal sprint trials (~70% of maximal sprint velocity) interspersed by 2 minutes of passive recovery. Finally, session-RPE (s-RPE) was assessed in every training session (45 sessions) across the 8 weeks.

**TABLE 1:** Sprint and jump training program for both groups* during the period of the study.

	Sessions 1–4		Sessions 5–9		Sessions 10–12	

	Sets	Reps	Sets	Reps	Sets	Reps
Bilateral hurdle jumps	3	6	4	5	3	5
Unilateral horizontal jumps	3R 3L	6 6	4R 4L	5 5	3R 3L	5 5
Vertical drop jumps	4	6	4	6	4	4
Horizontal drop jumps	2	6	2	6	2	4
Linear sprint	6	1 × 10-m	6	1 × 15-m	3	1 × 10-m
90º COD sprint	2R 2L	1 × 10-m 1 × 10-m	2R 2L	1 × 15-m 1 × 15-m	1R 1L	1 × 10-m 1 × 10-m

* Both groups performed the same exercises on sand or grass surfaces. COD = change of direction; R = right side; L = left side; Reps = repetitions. Recovery time between repetitions and sets were 15–30 s and 1–3 min, respectively.

**TABLE 2:** Typical weekly training schedule of the soccer players during the 8 weeks of intervention.

Monday	Tuesday	Wednesday	Thursday	Friday	Saturday	Sunday
Resistance training (45-min)	Technical-tactical training (50-min)	Sand or grass sprint-jump training (20–30-min)	Passing, shooting, and dribbling skills (40-min)	Technical-tactical training (40-min)	Friendly match	Rest
Small-sided games (60-min)		Offensive and defensive game situations (50-min)	Low demand (regenerative) activity (30-min)			

*Note:* resistance training involved traditional strength exercises (e.g., bench press, half-squat, hip-thrust, and prone row) performed with moderate loads (e.g., 40–60% of one-repetition maximum).

### Procedures

#### Vertical Jumping Tests

Vertical jump height was assessed using the SJ and CMJ. In the SJ, athletes were required to remain in a static position with a 90° knee flexion angle for ~2 s before jumping, without executing any preparatory movement. In the CMJ, athletes were instructed to execute a downward movement followed by complete extension of the legs and were free to determine the countermovement amplitude to avoid changes in jumping coordination [19]. All jumps were performed with the hands on the hips, with athletes being instructed to jump as high as possible. The jumps were performed on a contact platform (Elite Jump, S2 Sports, São Paulo, Brazil) and jump height was automatically calculated based on the flight time. A total of three attempts were allowed for each jump, interspersed by 15-s intervals. The best attempts for the SJ and CMJ were used for subsequent analyses.

#### Linear Sprinting Speed Test

Three pairs of photocells (Elite Speed, S2 Sports, São Paulo, Brazil) were positioned at the starting line and at the distances of 10 m and 17 m. Soccer players sprinted twice, starting from a standing position 0.5 m behind the starting line. The sprint tests were performed on an outdoor field of natural grass. Sprint velocity was calculated as the distance travelled over a measured time interval. The acceleration ability in the different distances (i.e., 0–10 m and 10–17 m) was calculated as the rate of change of velocity with respect to time. A 5-min rest interval was allowed between the two attempts and the fastest time was retained for analyses.

#### Curve Sprint Test

The CS test was performed as previously described [16]. The trajectory of the CS was the semi-circle of the goalkeeper area (of an official soccer field), which is standardized as follows: a 9.15-m radius (from the penalty spot); a 14.6-m distance from the initial to the final point in a straight line; an angle of 105.84º of amplitude from the penalty spot; a 17-m total distance (obtained from a trigonometrical analysis). The test was completed outdoors, on natural turf. Two pairs of photocells (Elite Speed, S2 Sports, São Paulo, Brazil) were positioned at the beginning and at the end of the curved trajectory. Soccer players sprinted twice for each side, and the fastest times from the right and left sides were retained.

#### Modified Zigzag Change of Direction Speed Test

The modified Zigzag COD test consisted of four 4.25-m sections (total linear distance of 17 m, to match the linear sprint and curve sprint distances) marked with cones set at 100° angles [16], requiring the athletes to decelerate and accelerate as fast as possible around each cone. Two maximal attempts were performed, with a 5-min rest interval between attempts. Starting from a standing position 0.5 m behind the first pair of timing gates (Elite Speed, S2 Sports, São Paulo, Brazil), athletes were instructed to complete the test as quickly as possible, until crossing the second pair of timing gates, placed 17 m from the starting line. The measurement was performed on an outdoor field of natural grass. The fastest time from the two attempts was retained.

### Session Rating of Perceived Exertion

The internal training load was recorded using the s-RPE method [18]. Approximately 30 min after completion of each training session, players were required to report the intensity of the whole session by means of a 10-point rating of perceived exertion scale [18]. This value was multiplied by the respective total duration of each training session. Daily s-RPE values for each group were used for the analyses.

### Statistical Analyses

Data are presented as mean ± standard deviation (SD). Data normality was checked using the Shapiro-Wilk test. Absolute and relative reliability of the data were determined using the coefficient of variation (CV) and a two-way random effects model intraclass correlation coefficient (ICC), based on the multiple attempts performed at pre-test. A two-way ANOVA with repeated measures (group*time interaction) followed by Tukey’s post-hoc was used to examine pre- and post-test differences between groups. The statistical level of significance was set as *P* < 0.05. Additionally, to determine the magnitude of pre- and post-changes, effect sizes (ES) along with 95% confidence intervals (CI) were calculated and interpreted using the thresholds proposed by Rhea [20] for highly trained subjects, as follows: < 0.25, 0.25–0.50, 0.50–1.00, and > 1.00 for trivial, small, moderate, and large, respectively.

## RESULTS

**FIG. 1:**
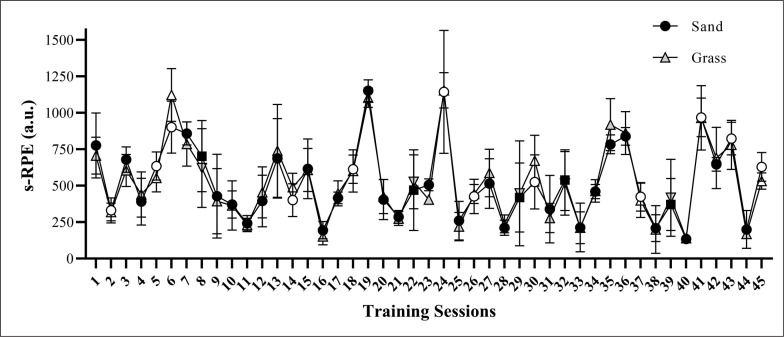
Variations in the session-rating of perceived exertion (s-RPE) across the 45 sessions analysed during the 8-week training period. Black circles and grey triangles represent technical and tactical training sessions; white symbols correspond to sand and grass training sessions; squares and inverted triangles represent friendly matches.

The CV values for all tests performed were < 5%. The ICC values for SJ, CMJ, 10-m and 17-m linear sprint, CS, and COD test were 0.98, 0.99, 0.88, 0.92, 0.84, and 0.82, respectively. No between-group significant differences were found for any variables tested in the baseline measures (*P* values ranging from 0.361 to 0.998). Figure 1 shows the variations in the s-RPE over the 8-week training period. Sand and grass groups showed similar changes in the s-RPE over the 45 sessions analysed (*P* = 0.223 for group*time interaction). Table 3 presents the comparisons of the vertical jump performances between pre- and post-assessments for both training groups. No significant changes were noted for SJ and CMJ height for both training groups (ES ranging from 0.16 to 0.57; *P* > 0.05).

**TABLE 3:** Comparisons of the vertical jump performances between pre- and post-assessments for both training groups.

	Groups	Pre	Post	ES (95% CI)	*P*-values	
					Pre *vs*. Post	Group*time interaction
**SJ (cm)**	Sand	37.5 ± 3.6	37.7 ± 4.3	0.08 (-0.66; 0.82)	0.995	0.661
	Grass	39.1 ± 3.6	38.8 ± 3.5	0.16 (-0.54; 0.86)	0.978	

**CMJ (cm)**	Sand	38.7 ± 4.1	40.0 ± 4.4	0.57 (-0.26; 1.35)	0.314	0.479
	Grass	41.5 ± 4.1	42.1 ± 4.4	0.39 (-0.35; 1.10)	0.823	

ES: effect size; CI: confidence intervals; SJ: squat jump; CMJ: countermovement jump.

**FIG. 2:**
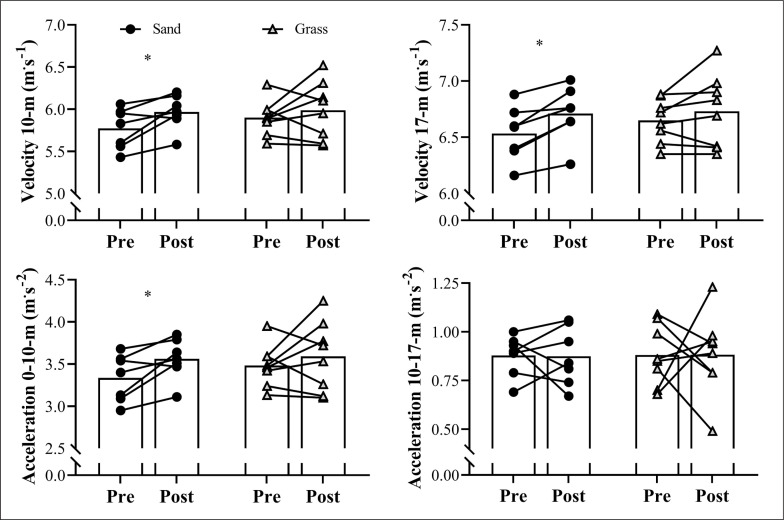
Variations in the 10- and 17-m linear sprint velocities and in the 0–10- and 10–17-m acceleration capacities in the sand and grass groups during the 8-week training period. Symbols represent individual results and bars correspond to mean values. *Main effect of time (*P* < 0.05) in comparison to “Pre” assessments.

Figure 2 depicts the changes in sprint velocity and acceleration ability across the 8 weeks, for both groups. Significant increases were observed for the sand group in 10-m (*P* = 0.05; ES [95% CI] = 1.16 [0.15; 2.11]) and 17-m (*P* = 0.025; ES [95% CI] = 1.81 [0.54; 3.03]) linear sprint velocity, and in 0–10-m acceleration (*P* = 0.023; ES [95% CI] = 1.15 [0.10; 2.10]) after the training intervention. Meanwhile, no significant change was noted in the 10–17-m acceleration for the sand group (*P* = 0.963; ES [95% CI] = 0.03 [-0.77; 0.71]). No significant differences were observed for 10-m (*P* = 0.324; ES [95% CI] = 0.30 [-0.42; 1.00]) and 17-m (*P* = 0.405; ES [95% CI] = 0.47 [-0.28; 1.19]) linear sprint velocities and acceleration abilities in 0–10 m (*P* = 0.311; ES [95% CI] = 0.31 [-0.41; 1.01]) and 10–17 m (*P* = 0.989; ES [95% CI] = 0.01 [-0.69; 0.70]) in the grass group when comparing pre- and post-tests. No group*time interaction was detected for 10- and 17-m linear sprint performance (*P* = 0.548 and 0.21, respectively) and acceleration abilities in 0–10 m and 10–17 m (*P* = 0.453 and 0.966, respectively).

**FIG. 3:**
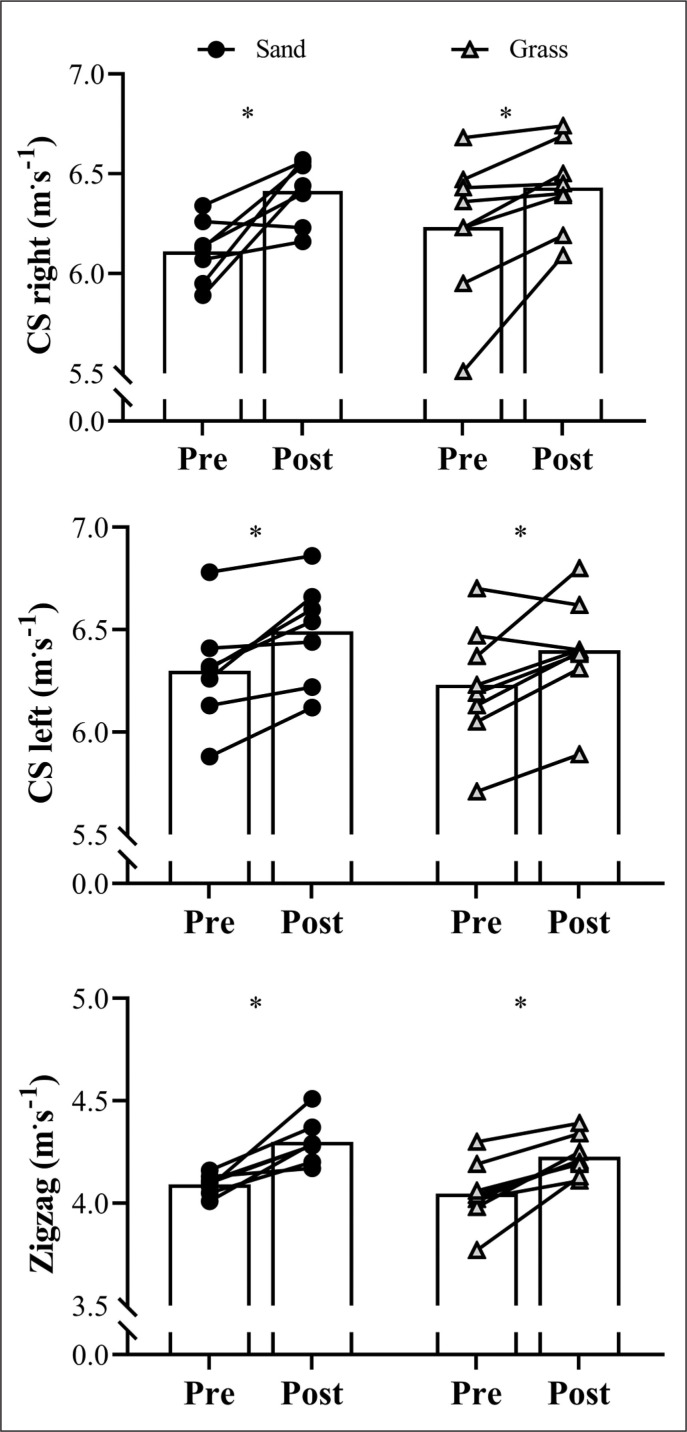
Variations in the curve sprint for right and left sides, and Zigzag change of direction velocities in the sand and grass groups during the 8-week training period. Symbols represent individual results and bars correspond to mean values. *Main effect of time (*P* < 0.05) in comparison to “Pre” assessments.

Figure 3 shows the comparisons of CS and COD performances between pre- and post-assessments for both training groups. Both sand and grass groups revealed similar increases in the CS velocity for both right (*P* = 0.002; ES [95% CI] = 1.28 [0.23; 2.27]; *P* = 0.019; ES [95% CI] = 1.10 [0.18; 1.97], for sand and grass groups, respectively) and left sides (*P* = 0.006; ES [95% CI] = 1.46 [0.34; 2.53]; *P* = 0.009; ES [95% CI] = 0.98 [0.10; 1.81], for sand and grass groups, respectively) as well as for the COD velocity (*P* < 0.001; ES [95% CI] = 1.72 [0.49; 2.90]; *P* = 0.002; ES [95% CI] = 1.93 [0.71; 3.12], for sand and grass groups, respectively) after the 8-week training period. No group*time interaction was noted for CS and Zigzag COD velocities (*P* = 0.354 and 0.756 for right and left sides, respectively; and *P* = 0.615 for Zigzag).

## DISCUSSION

This study examined and compared the effects of two different mixed training programmes (i.e., plyometric and sprint training strategies performed on sand or grass surfaces) on the jump- and speed-related performance of elite young soccer players across an 8-week inter-season training period. Our main results revealed that: 1) similar training loads (i.e., assessed by s-RPE) were observed between both experimental groups during the 8 weeks; 2) no improvements in vertical jump performance were found in either of the groups; 3) linear sprint velocity and acceleration were significantly enhanced after sand training; 4) both training strategies significantly increased CS and COD speeds. These data confirm the effectiveness of mixed training strategies (i.e., sprint-jump training) performed on either sand or grass surfaces for inducing positive changes in speed-related performance. Nevertheless, the sand surface appears to be more efficient to simultaneously develop a range of different speed capacities (i.e., linear sprint, CS, and COD speed) in elite young soccer players.

The s-RPE values varied similarly in both groups over 45 training sessions. Considering that the s-RPE responses are influenced by distinct physical and physiological parameters (e.g., speed endurance and aerobic fitness) [21–23], this result suggests that the randomization process was well conducted and balanced, allocating players with similar fitness levels and demands to both groups. Importantly, the s-RPE values of both groups were similar for soccer training sessions and friendly matches, and when comparing sand and grass training sessions. The latter finding is especially relevant, since previous studies reported that training on sand is associated with greater metabolic energy cost [9] and lactate accumulation [24], compared to harder surfaces, which, theoretically, may result in higher perceived training loads. However, the high-speed impacts and high application of eccentric forces against hard surfaces may be more demanding for the soccer players, which, in turn, could equalize and balance the overall training stimulus, leading athletes from both groups to report similar perceived training loads after the sprint-jump training sessions. In fact, previous studies have shown that performing plyometrics on harder surfaces results in higher levels of muscle soreness and indirect markers of muscle damage (e.g., creatine kinase activity) compared to sand surfaces [8, 10]. Therefore, the RPE after training sessions executed either on sand or hard surfaces may be influenced by different factors but result in similar RPE responses. Further studies are necessary to clarify the pathways and mechanisms behind these “similar perceptions” and responses to training.

Contrary to previous investigations [6, 8], we did not find significant improvements in vertical jumping ability after sand or grass interventions. To some extent, this result may be explained by two complementary factors: 1) twelve sprint-jump training sessions were completed over 8 weeks by both groups, which means that, across some weeks, only one plyometric/speed training session was performed; and 2) the total volume of jumps executed during the entire training phase was lower than that reported or indicated in previous studies (~110 jumps per week in 12 sessions vs > 140 jumps per week in > 14 sessions) [25, 26]. Indeed, during periods with high volumes of technical-tactical training and matches, higher frequencies and volumes of plyometric exercises seem to be necessary to substantially improve jumping performance [25–27]. For example, Ramirez-Campillo et al. [28] found only small increases in the CMJ height (4.3% versus 2.2% in the control group) of young soccer players, after examining the effects of a 7-week low-volume plyometric training (i.e., 60 drop jumps per session, twice a week) programme implemented during the in-season phase. It is worth noting that the regular training routine of these players (according to the authors) comprised “usual soccer sessions” with a much shorter duration than that described in our study (90 min vs. 134 min, on average, in our study). Unquestionably, this higher volume of soccer-specific training along with the inadequate time for optimizing power-related capabilities may further compromise the proper development of jumping ability. In this regard, soccer strength and conditioning coaches have already stated that the main challenges faced during the preparation of modern soccer players are associated with the difficulties imposed by the congested fixture [4] schedules, which hampers the balance between strength and conditioning practices (e.g., strength-power training) and soccer-specific training. In fact, when asked if they would change anything in their training routines, given unlimited time and resources, the most frequent responses of these practitioners were related to increases in the frequency and volume of neuromuscular training strategies [4, 5]. Of note, the plyometric programmes used in this study were defined in accordance with the technical staff of the club and were adequately implemented within a real training scenario. This should be considered in future interventions with soccer players, especially when improvements in vertical jump performance are expected.

Maximum acceleration capacity (i.e., 0–10 m) and linear sprint velocity improved only in the sand training group. Similar results were obtained by Impellizzeri et al. [8], who compared the effects of a 4-week plyometric training programme executed on sand or grass surfaces in amateur soccer players, in which linear sprint velocity increased significantly only in the sand group. Although the mechanisms underlying this result remain to be fully elucidated, it could be speculated that the use of sand exercises may provide a greater stimulus to muscle contractile properties (in comparison with grass drills) [6, 29, 30]. Indeed, to perform explosive drills on sand (e.g., maximal sprints and jumps), soccer players are forced to overcome the resistance naturally generated by this soft surface, which results in increased contraction time and greater displacement of the lower extremity joints and, consequently, in more work being done by the muscles [31, 32]. Likewise, during the initial phases of sprinting (i.e., acceleration phase), athletes have to accelerate rapidly to overcome the inertia of the body mass, and thus achieve higher velocities [33]. In practical terms, these movements tend to resemble those observed during sand training that rely heavily on the application of substantial amounts of force onto the ground, with a marked and prominent contribution of concentric muscle contractions [6, 8, 30]. At least in theory, the superior gains elicited by sand surfaces in both acceleration and linear sprint velocity may be related to these biomechanical and neuromuscular factors and similarities that can lead to a greater participation (and thus increase) of concentric strength during the initial phases of sprinting [6] (i.e., maximum acceleration phase). This hypothesis should be tested in future research, specifically designed to examine the neuromechanical adaptations to soft and hard surfaces.

After the 8-week training period, both groups presented significant (and similar) increases in CS velocity. This suggests that the mixed training programme used in our study, comprising unilateral and bilateral vertical and horizontal jumps of different types (e.g., drop jumps and hurdles jumps), short linear sprints, and 90º COD sprint drills, was effective to improve curvilinear sprint performance, irrespective of training surface (i.e., sand or grass). Curiously, unlike linear sprint velocity, which increased only in the sand training group, CS performance improved significantly in both groups. Although seemingly similar and largely correlated (r ≥ 0.74 for linear and curvilinear sprints of equal distances, i.e., 17 m) [19], linear sprint and CS abilities exhibit some critical differences. In general, curvilinear running is associated with longer contact times and greater muscle activation in the gastrocnemius medialis, and lower muscle activation in the biceps femoris, compared to traditional sprints [17, 34]. In addition, over curved paths, the “inside leg” is usually more affected than the “outside leg”, exhibiting higher activation of hip internal rotation muscles (i.e., semitendinosus and adductor) and longer foot contact times, whereas the outside leg displays higher activation of hip external rotation muscles (i.e., biceps femoris and gluteus medius) [17, 34]. Huge discrepancies in muscle activation and mechanical and technical aspects are also observed when comparing different jump types [35] and sprint stimuli [17]. Therefore, it can be speculated that the mixed training strategy (Table 1) proposed in our study contributed to promoting positive adaptations in certain mechanisms underpinning CS performance. However, we recognize that this is simply speculation, which should be further discussed and clarified.

Similarly to CS, COD sprint velocity increased as a result of both training interventions. This result agrees with previous research comparing the effects of sand and hard surface training interventions on the COD ability of team-sport athletes and supports our initial assumption that the combination of a range of different plyometric and (multi-directional) sprint activities would result in improved speed-related performance [36]. These beneficial effects may be explained, at least in part, by the reasons and mechanisms mentioned above, thus being a product of improvements in a series of neuromechanical factors directly related to COD performance (e.g., deceleration capacity, inter-limb muscle coordination, and motor-unit activation) [37–39]. Again, it should be emphasized that such improvements are commonly found after short-term training programmes (i.e., ≤ 8 weeks) involving sprint and/or jump drills [40–42], which reinforces our argument regarding their potential transference to COD performance.

In summary, we found that the mixed training strategy used in our study, comprising a variety of sprint and jump drills, was able to improve CS and COD speed performances, irrespective of training surface (i.e., sand or grass). Maximum acceleration capacity (i.e., 0–10 m) and linear sprint velocity (i.e., ≤ 17 m) increased only in the sand group, indicating the effectiveness of a sand surface to promote significant gains in short-sprint performance. The low volume of plyometric training (compared to the high volume of soccer-specific training) may potentially explain the lack of improvements reported for vertical jumping ability, for both groups. This study is limited by the small sample size and the lack of a pure control group of subjects who did not participate in any of the interventions, which is commonplace in studies involving elite team-sport players during the competitive season. Under this rationale, it is important to evaluate the extent to which the soccer-specific training content, and the other stimuli present in their weekly schedules (i.e., resistance training), could influence the physical performance of soccer players across the 8 weeks. Lastly, we did not assess the neuromuscular mechanisms and the biomechanical adjustments involved in the performance changes observed. Hence, it would be interesting to examine whether there are differences in neuromechanical adaptations after training programmes executed on different surfaces and compare the effects of sand training with those resulting from training strategies which also lead to acute decreases in sprint velocity by the addition of external resistance (e.g., sled towing).

## CONCLUSIONS

We compared the effects of two different training surfaces across an 8-week training period in elite young soccer players. Our results reinforce the findings of a recent meta-analysis [6], supporting the use of a sand surface as an efficient and practical strategy to improve the speed-related performance of team-sport athletes. Moreover, sand training was not perceived as more intense than grass training, which may have important implications for training prescription, especially during congested match schedules and when carefully balanced. Overall, the mixed-training scheme implemented in our study, performed on both sand and grass surfaces, significantly improved (and to a similar extent) CS and COD speed performances. On the other hand, maximum acceleration capacity and linear sprint velocity increased only in the sand group. Bearing in mind the importance of acceleration and sprint capabilities in elite soccer [1–3], we consider that our results may be highly informative and practically relevant for soccer coaches and sport scientists.

## CONFLICT of interest statement

The authors declare no conflict of interest.
